# Widespread effect of *N*-acetyl-d-glucosamine assimilation on the metabolisms of amino acids, purines, and pyrimidines in *Scheffersomyces stipitis*

**DOI:** 10.1186/s12934-018-0998-4

**Published:** 2018-09-25

**Authors:** Kentaro Inokuma, Mami Matsuda, Daisuke Sasaki, Tomohisa Hasunuma, Akihiko Kondo

**Affiliations:** 1 Graduate School of Science, Technology and Innovation, Kobe University, 1-1 Rokkodai-cho, Nada-ku, Kobe, Hyogo 657-8501 Japan; 2 Biomass Engineering Program, RIKEN, 1-7-22 Suehiro-cho, Tsurumi-ku, Yokohama, Kanagawa 230-0045 Japan

**Keywords:** Metabolome analysis, *N*-acetylglucosamine, Glucose, Xylose, *Scheffersomyces stipitis*, Non-conventional yeast

## Abstract

**Background:**

Following cellulose, chitin is the most abundant renewable resource and is composed of the monomeric amino sugar *N*-acetyl-d-glucosamine (GlcNAc). Although many yeasts, including *Saccharomyces cerevisiae*, have lost their ability to utilize GlcNAc, some yeasts are able to use GlcNAc as a carbon source. However, our understanding of the effects of GlcNAc on the intracellular metabolism of nitrogen-containing compounds in these yeast species is limited.

**Results:**

In the present study, we quantitatively investigated the metabolic responses to GlcNAc in the GlcNAc-assimilating yeast *Scheffersomyces stipitis* (formerly known as *Pichia stipitis*) using capillary electrophoresis time-of-flight mass spectrometry (CE-TOFMS). The comprehensive analysis of the metabolites extracted from *S. stipitis* cells grown in glucose, xylose, or GlcNAc revealed increased intracellular accumulation of a wide range of nitrogen-containing compounds during GlcNAc assimilation in this yeast. The levels of aromatic, branched-chain, and sulfur-containing amino acids and adenine, guanine, and cytosine nucleotides were the highest in GlcNAc-grown cells.

**Conclusions:**

The CE-TOFMS analysis revealed a positive effect for GlcNAc on the intracellular concentration of a wide range of nitrogen-containing compounds. The metabolomic data gathered in this study will be useful for designing effective genetic engineering strategies to develop novel *S. stipitis* strains for the production of valuable nitrogen-containing compounds from GlcNAc.

**Electronic supplementary material:**

The online version of this article (10.1186/s12934-018-0998-4) contains supplementary material, which is available to authorized users.

## Background

The amino sugar *N*-acetyl-d-glucosamine (GlcNAc) is the monomeric constituent of chitin, which is one of the most abundant renewable resources found in nature, second only to cellulose [1]. Chitinolytic organisms, such as marine and soil bacteria and fungi, degrade chitin into GlcNAc and then utilize it as their principal source of carbon and nitrogen [2–4]. On the other hand, many yeast species including the most widely used industrial yeast, *Saccharomyces cerevisiae*, have lost their ability to utilize GlcNAc [5] except for some dimorphic yeast species that separated early from the common yeast evolutionary trunk [6, 7].

In dimorphic yeasts such as *Candida albicans* and *Yarrowia lipolytica,* GlcNAc is known to be a potent inducer of morphological transition [8]. Numerous studies focused on the GlcNAc metabolic pathway in these yeasts and the effect of GlcNAc on their morphogenesis have been reported [7, 9–11]. However, few studies have evaluated the metabolic response to GlcNAc in these yeast strains [12], and no comprehensive analysis has been published concerning the influence of GlcNAc on the metabolism of nitrogen-containing compounds such as amino acids, purines, and pyrimidines in yeast cells. Therefore, influences of GlcNAc on carbon and nitrogen metabolisms in GlcNAc-assimilating yeasts are still poorly understood.

*Scheffersomyces stipitis* (formerly known as *Pichia stipitis*) is a Crabtree-negative, homothallic yeast, found mainly in haploid form [13]. *S. stipitis* has greater respiratory capacity and broader sugar utilization capacity than *S. cerevisiae* [14, 15] and is extensively studied for its capacity to ferment xylose to ethanol. Recently, we reported that *S. stipitis* uses GlcNAc as its sole carbon source even though this yeast is not dimorphic [5]. In fact, in our experiments, *S. stipitis* NBRC10063 (CBS6054) consumed almost all of the GlcNAc introduced (50 g/L) and produced ethanol with high yield [82% (mol ethanol/mol GlcNAc consumed)] under oxygen-limited conditions. However, the GlcNAc metabolic pathway of *S. stipitis* and its metabolic state during GlcNAc fermentation have not been identified.

In the present study, the metabolic responses to GlcNAc in *S. stipitis* were evaluated using metabolomic analysis via capillary electrophoresis time-of-flight mass spectrometry (CE-TOFMS). Metabolic profiling based on CE-TOFMS has emerged as one of the most powerful approaches for understanding complex biological systems [16] because it provides comprehensive and quantitative information concerning the charged metabolites from cellular extracts that reflects their metabolic state [17]. To our knowledge, this is the first time the metabolic effects of GlcNAc have been evaluated in *S. stipitis*.

## Methods

### Strains and media

*Scheffersomyces stipitis* NBRC10063 was obtained from the NITE Biological Resource Center (NBRC). Yeast cells were pre-cultured in 5 mL of Yeast extract peptone dextrose (YPD) medium [10 g/L yeast extract, 20 g/L Bacto-peptone (Difco Laboratories, Detroit, MI, USA), and 20 g/L glucose] in a shaker incubator (180 rpm at 30 °C; BR-43FL; Taitec, Saitama, Japan) for 18 h. The yeast cells were harvested by centrifugation at 1000×*g* for 5 min and then washed twice with distilled water. The washed cells were used for anaerobic fermentation as described below.

### Anaerobic fermentation of glucose, xylose, and GlcNAc

Ethanol fermentation of glucose, xylose, and GlcNAc (50 g/L in YP medium containing 10 g/L yeast extract, 20 g/L Bacto-peptone; YPD50, YPX50, and YPGN50, respectively) was anaerobically performed in closed 100 mL bottles equipped with a CO_2_ outlet. Washed yeast cells were inoculated in 20 mL of YPD50, YPX50, or YPGN50 media at an initial cell density (OD_600_) of 0.1. Fermentation was initiated by the addition of yeast cells into the fermentation medium, followed by rotation in a shaker incubator (180 rpm at 30 °C). The culture broth was sampled every 24 h for 4 days, and its OD_600_ was measured with a UV–VIS spectrophotometer (UVmini-1240, Shimadzu, Kyoto, Japan). The OD_600_ was then converted to dry cell weight (DCW; g/L) by multiplying the value by an experimentally determined coefficient (0.15). The concentrations of glucose, xylose, GlcNAc, ethanol, and acetate in each sample were determined using high-performance liquid chromatography (HPLC) (Shimadzu) as described previously [5]. Ammonia concentration in the culture medium was assessed using LabAssay™ Ammonia (Wako Pure Chemical Industries, Ltd., Osaka, Japan) according to the manufacturer’s protocol.

### Intracellular metabolite extraction and CE-TOFMS analysis

After fermentation in YPD50, YPX50, and YPGN50 media for 24 h as described above, 5 mL of the culture medium was sampled and the intracellular metabolites were extracted according to a previously reported method [18] with minor modifications. These modifications include the addition of 10 μL of 40 mM 1,4-piperazinediethanesulfonic acid (PIPES) and l-methionine sulfone to the samples as internal standards for the mass analysis of anionic and cationic species, respectively, as well as the use of a boiling ethanol method [19] for metabolite extraction at 95 °C for 5 min. The extracts were dried in a vacuum evaporator (CVE-3100, Tokyo Rikakikai, Osaka, Japan) overnight and stored at − 80 °C until use.

The dried metabolites were dissolved in ultrapure water and the anionic and cationic intermediate concentrations were measured using CE-TOFMS as described previously [20]. All the metabolites quantified in the CE-TOFMS analysis are listed in Additional file 1: Table S1.

### Quantitative real-time PCR (qRT-PCR)

Yeast cells harvested at 24 h of fermentation in YPD50, YPX50, and YPGN50 media were used for RNA preparation to observe the gene expression. The cells were homogenized using the Shake Master Neo (Bio Medical Science, Tokyo, Japan) and 0.5 mm glass beads. Total RNA was isolated from the homogenized cells using the NucreoSpin RNA (Macherey–Nagel, Düren, Germany) according to the manufacturer’s instructions. RNA concentration and quality were determined using a NanoDrop One spectrophotometer (Thermo Fisher Scientific Inc., Waltham, MA, USA) and an Agilent 2100 Bioanalyzer (Agilent Technologies, Palo Alto, CA, USA), respectively. cDNA was synthesized from 200 ng of total RNA using the RevarTra Ace qPCR RT Master Mix with gDNA Remover (TOYOBO, Osaka, Japan). Expression levels were quantified using a KOD SYBR qPCR Mix (TOYOBO) in the Mx3005P RT-PCR platform (Agilent Technologies). Amplifications were performed under the following conditions: 98 °C for 2 min; 40 cycles of 98 °C for 10 s, 60 °C for 10 s, and 68 °C for 30 s. A melting analysis was conducted at the end of the amplification cycle to verify the specificity of the reaction. Gene expression levels of target genes were normalized to that of the housekeeping gene *TUB2*. Primers used for qRT-PCR are listed in Additional file 2: Table S2.

### Statistical analyses

The data are presented as the mean of three independent replications. Significant differences between culture conditions were calculated according to paired comparisons using Student’s *t*-tests using MS Excel 2011. A *p* < 0.05 was considered statistically significant. A principal component analysis (PCA) was conducted using JMP software version 13.0 (SAS Institute Cary, NC, USA).

## Results

### Anaerobic fermentation of glucose, xylose, and GlcNAc

**Fig. 1:**
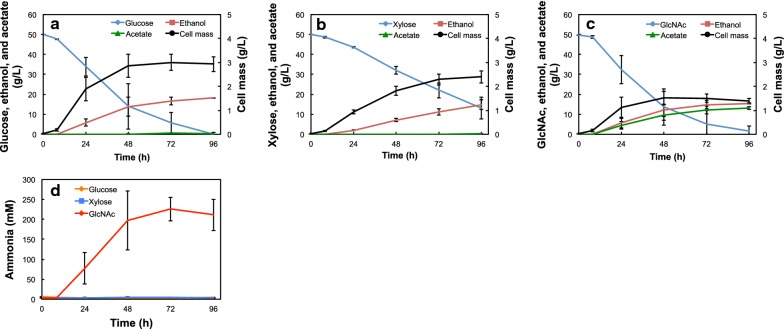
Time-course of anaerobic fermentation of **a** glucose, **b** xylose, and **c** GlcNAc by *S. stipitis* NBRC10063 at 30 °C. **d** Indicates ammonia concentration in each culture condition. The yeast cell mass is indicated in dry cell weight (DCW). Data are presented as the means ± standard deviation (n = 3)

To determine the effects of GlcNAc on cell growth and fermentation products of in *S. stipitis*, anaerobic fermentation of GlcNAc and conventional carbon sources (glucose and xylose) was performed. The *S. stipitis* NBRC10063 strain was cultivated in YP media containing 50 g/L of glucose (YPD50), xylose (YPX50), or GlcNAc (YPGN50) anaerobically for 96 h (Fig. 1 and Table 1). This yeast produced 18.2, 14.8, and 15.5 g/L of ethanol from glucose, xylose, and GlcNAc during this time period, respectively. Furthermore, in YPGN50, the levels of acetate (13.1 g/L, which corresponds to 218 mM) and ammonia (211 mM) were almost equal to the molar concentration of consumed GlcNAc (217 mM) at 96 h (Fig. 1c, d). Because of the production of these byproducts, the conversion factors of cell mass (*Y*_*X/S*_) and ethanol production (*Y*_*P/S*_) after 96 h of fermentation in the YPGN50 medium were significantly lower than those calculated for the other media (Table 1). Based on the result shown in Fig. 1, we decided to use the intracellular metabolites extracted after 24 h of cultivation for the following metabolomic analysis.

**Table 1:** Anaerobic fermentation of glucose, xylose, and GlcNAc by *S. stipitis* NBRC10063 for 96 h

Carbon sources	Ethanol (g/L)	Acetate (g/L)	Ammonia (mM)	DCW (g/L)	*Y*_*X/S*_ (g DCW/g substrate)	*Y*_*P/S*_ (g ethanol/g substrate)
Glucose	18.2 ± 0.2	0.5 ± 0.4	4.4 ± 1.0	2.93 ± 0.31	0.059 ± 0.006	0.365 ± 0.004
Xylose	14.8 ± 2.4	0.2 ± 0.2	3.1 ± 0.5	2.40 ± 0.26	0.065 ± 0.003	0.399 ± 0.006
GlcNAc	15.5 ± 0.2	13.1 ± 0.6	211.5 ± 39.3	1.39 ± 0.09	0.029 ± 0.001	0.323 ± 0.018

The averages for three independent experiments are shown with their standard deviations

### Intracellular metabolite profile

To investigate the metabolic response of *S. stipites* to GlcNAc, the intracellular metabolites were extracted after 24 h of cultivation under anaerobic conditions in YPD50, YPX50, and YPGN50 media, and metabolomic analysis was performed using CE-TOFMS. The metabolites verified in our CE-TOFMS analysis (> 130 in total) are listed in Additional file 1: Table S1. Of these, 106 compounds associated with carbon and nitrogen metabolism were detected in at least one sample from at least one culture condition.

**Fig. 2:**
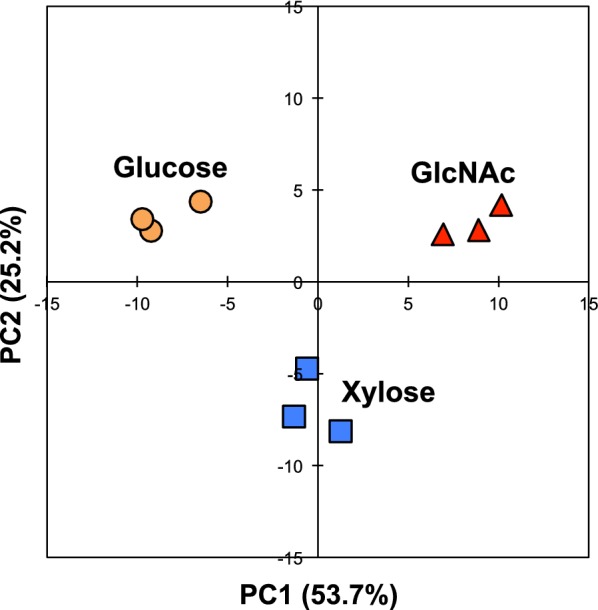
Principal component analysis (PCA) of the metabolomic data for *S. stipitis* NBRC10063 at 24 h after the initiation of ethanol fermentation with three different carbon sources (glucose, xylose, or GlcNAc). Data plots for individual samples cultivated with glucose, xylose, and GlcNAc are indicated in orange, blue, and red, respectively

To reduce data dimensionality and obtain an overview of the metabolite profile, a principal component analysis (PCA) was performed using data sets of individual samples obtained by the CE-TOFMS analysis. The PCA revealed that the two-dimensional plots of principal component 1 (PC1) and PC2 segregate the data cluster along the carbon sources (Fig. 2). The plots of PC1 axis for *S. stipitis* cultivated with glucose, xylose, and GlcNAc was clearly separated and the plots of PC2 axis for the yeast cultivated with xylose was also separated from the other two groups. The proportion of variance, which shows how much of the original data is retained by each principal component, of PC1 and PC2 were 53.7% and 25.2%, respectively. Metabolites contributing to PC1 and 2 were then extracted based on their factor loading scores (Table 2 and Additional file 1: Table S1). Interestingly, 13 of the top 20 metabolites contributed to PC1 were nitrogen-containing compounds and include not only intermediates of the GlcNAc metabolic pathway but also amino acids, purines, and pyrimidines. In addition, phosphate, lactate, and citrate also highly contribute to PC1. Alternatively, pentose phosphate pathway components and uracil nucleotides (UMP, UDP, and UTP) were included in the metabolites contributing to PC2.

**Table 2:** The top 20 metabolites with highest factor loadings corresponding to PC1 and PC2

PC1		PC2	
Metabolites	Factor loadings	Metabolites	Factor loadings
Nicotinate	0.9831	UDP	0.9853
3-Hydroxybutyrate	0.9761	UTP	0.9672
Guanosine	0.9747	Ru5P (Xu5P)^a^	0.9669
CMP	0.9643	GAP	0.9491
*cis*-Aconitate	0.9586	ADP-d-ribose	0.8924
Adenosine	0.9527	UMP	0.8880
Citrate	0.9511	UDP-d-glucose	0.8805
GlcN6P	0.9502	Guanine	0.8671
GSSG	0.9474	S7P	0.8644
Phosphate	0.9473	l-Arginine	0.7588
Lactate	0.9454	F1,6BP	0.7402
GlcN1P	0.9438	G1P	0.7316
GlcNAc	0.9424	dTDP	0.6788
Cytidine	0.9419	AMP	0.6780
GlcNAc6P	0.9407	l-Lysine	0.6661
Pyruvate	0.9390	d-Glyceric acid	0.6497
Tyramine	0.9277	NADH	0.6413
PEP	0.9242	ADP	0.6139
l-Phenylalanine	0.9202	GMP	0.5789
GDP	0.9140	Hypoxanthine	0.5645

^a^Ru5P and Xu5P are epimers and could not be distinguished via CE-TOFMS analysis in this study

### GlcNAc metabolic pathway and central metabolism

**Fig. 3:**
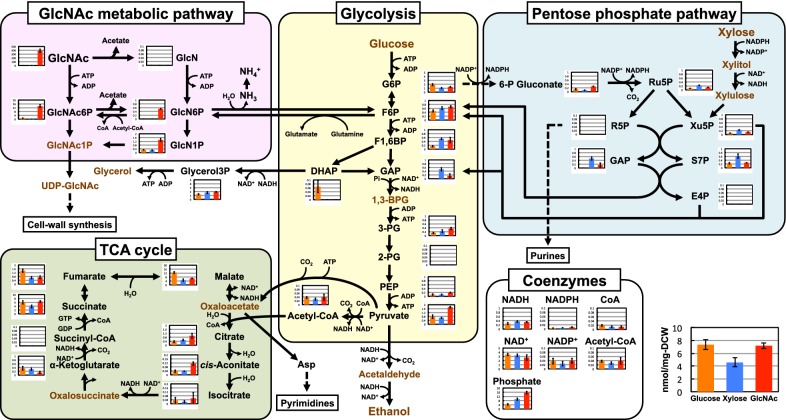
Concentration (nmol/mg DCW) of metabolites involved in the GlcNAc metabolic pathway and central metabolism in *S. stipitis* NBRC10063 after fermenting glucose (orange bars), xylose (blue bars), or GlcNAc (red bars) for 24 h. Arrows indicate the main direction of metabolic flow. Dashed arrows indicate reactions involving more than one enzymatic step. Metabolites written in brown were not analyzed in this study. Data are presented as the means ± standard deviation (n = 3). 1,3-BPG: 1,3-bisphosphoglycerate; 2-PG: 2-phosphoglycerate; 3-PG: 3-phosphoglycerate; 6-P gluconate: 6-phosphogluconate; DHAP: dihydroxyacetone phosphate; E4P: erythrose-4-phosphate; F1,6BP: fructose-1,6-bisphosphate; F6P: fructose-6-phosphate; G3P: glyceraldehyde-3-phosphate; G6P: glucose-6-phosphate; GlcN: glucosamine; GlcN1P: glucosamine-1-phosphate; GlcN6P: glucosamine-6-phosphate; GlcNAc: *N*-acetyl-d-glucosamine; GlcNAc1P: *N*-acetyl-d-glucosamine-1-phosphate; GlcNAc6P: *N*-acetyl-d-glucosamine-6-phosphate; Glycerol3P: glycerol-3-phosphate; NAD^+^: nicotinamide adenine dinucleotide; NADP^+^: nicotinamide adenine dinucleotide phosphate; PEP: phosphoenolpyruvate; Ru5P: ribulose-5-phosphate; R5P: ribose-5-phosphate; S7P: sedoheptulose-7-phosphate; UDP-GlcNAc: uridine diphosphate-*N*-acetyl-d-glucosamine; Xu5P: xylulose-5-phosphate

Using the metabolomic data, the states of the GlcNAc, its derivatives, and central metabolism in *S. stipitis* were investigated (Fig. 3). In GlcNAc-containing medium, we observed GlcNAc, GlcNAc-6-phosphate (GlcNAc6P), glucosamine-6-phosphate (GlcN6P), and glucosamine-1-phosphate (GlcN1P) accumulation. However, no glucosamine (GlcN) was detected under any conditions. The large amount of GlcNAc (388.6 ± 43.6 nmol/mg DCW) detected in GlcNAc-grown cells likely includes extracellular GlcNAc.

Many glycolytic metabolites, such as fructose-6-phosphate (F6P), fructose-1,6-bisphosphate (F1,6BP), 3-phosphoglycerate (3-PG), phosphoenolpyruvate (PEP), and pyruvate, were also increased in GlcNAc-grown cells (Fig. 3). On the other hand, in the cells grown in xylose, metabolites involved in the non-oxidative phase of the pentose phosphate pathway, such as ribulose-5-phosphate (Ru5P), xylulose-5-phosphate (Xu5P), sedoheptulose-7-phosphate (S7P), and glyceraldehyde-3-phosphate (GAP), exhibited the highest levels.

Among the metabolites associated with the tricarboxylic acid (TCA) cycle, citrate and *cis*-aconitate concentration was higher in the GlcNAc-grown cells than it was in the glucose- or xylose-grown cells, while the levels of malate, fumarate, and α-ketoglutarate in GlcNAc-grown cells was lower than that in the glucose-grown cells (Fig. 3).

### Amino acids and their related nitrogen-containing compounds

**Fig. 4:**
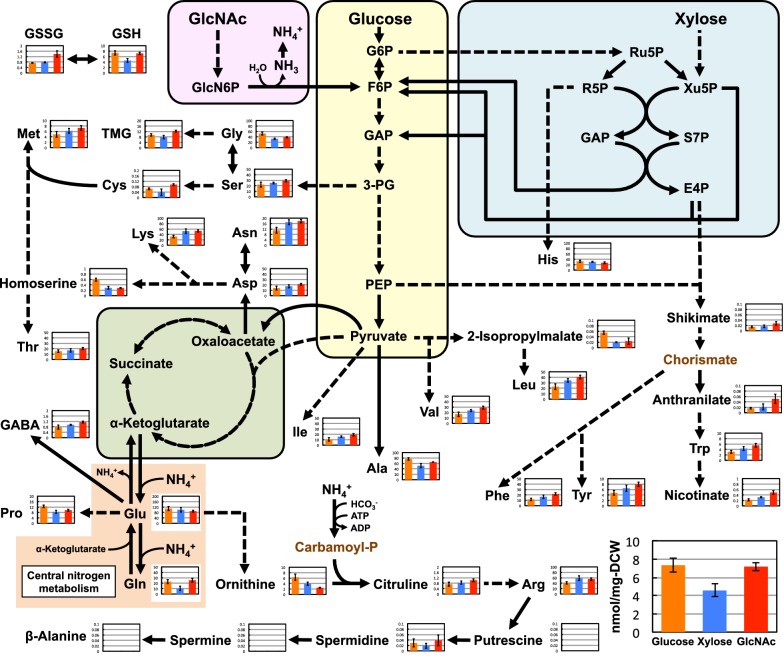
Concentration (nmol/mg DCW) of amino acids and related nitrogen-containing compounds in *S. stipitis* NBRC10063 after fermenting glucose (orange bars), xylose (blue bars), or GlcNAc (red bars) for 24 h. Arrows indicate the main direction of metabolic flow. Dashed arrows indicate reactions involving more than one enzymatic step. Metabolites written in brown were not analyzed in this study. Data are presented as the means ± standard deviation (n = 3). *Ala* alanine, *Arg* arginine, *Asn* asparagine, *Asp* aspartate, *Carbamoyl-P* carbamoyl phosphate, *Cys* cysteine, *GABA* γ-aminobutyrate, *Gln* glutamine, *Glu* glutamate, *Gly* glycine, *GSH* reduced glutathione, *GSSG* oxidized glutathione, *His* histidine, *Ile* isoleucine, *Leu* leucine, *Lys* lysine, *Met* methionine, *Phe* phenylalanine, *Pro* proline, *Ser* serine, *Thr* threonine, *TMG* trimethylglycine, *Trp* tryptophan, *Tyr* tyrosine, *Val* valine

The metabolomic data was also used to investigate the metabolic states of amino acids and related nitrogen-containing compounds in *S. stipitis* (Fig. 4). Among the 20 common proteinogenic amino acids, 13 (valine, threonine, cysteine, isoleucine, leucine, asparagine, aspartate, lysine, methionine, arginine, phenylalanine, tyrosine, and tryptophan) were found at higher concentrations in the GlcNAc-grown cells than those in the glucose-grown cells. In contrast, three amino acids (glycine, alanine, and proline) showed the opposite trend, being more concentrated in the glucose-grown cells compared to the GlcNAc-grown cells, while no significant differences were observed in the levels of the other four amino acids (serine, glutamine, glutamate, and histidine). Compared with those in the other conditions, the levels of some non-proteinogenic amino acids [anthranilate, citrulline, γ-aminobutyrate (GABA)] and nitrogen-containing compounds derived from amino acids [trimethylglycine (TMG) and nicotinate] were also increased in the cells grown in GlcNAc. Furthermore, the total glutathione levels in the yeast grown in glucose and GlcNAc were similar, while the ratio of oxidized glutathione (GSSG)-to-reduced glutathione (GSH) in the GlcNAc-grown cells was approximately twofold higher than that in glucose-grown cells.

### Purines and pyrimidines

**Fig. 5:**
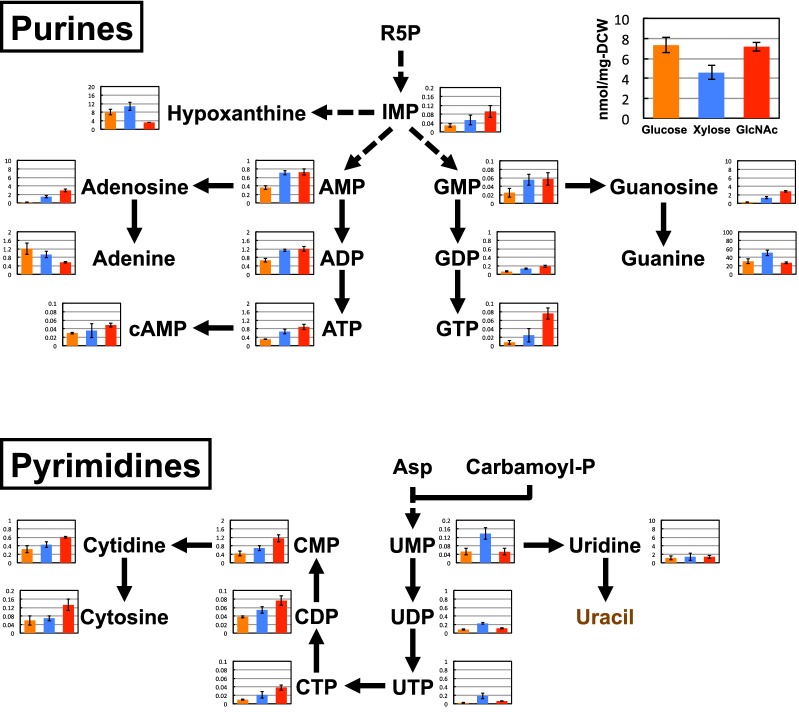
Concentration (nmol/mg DCW) of purines and pyrimidines in *S. stipitis* NBRC10063 after fermenting glucose (orange bars), xylose (blue bars), or GlcNAc (red bars) for 24 h. Arrows indicate the main direction of metabolic flow. Dashed arrows indicate reactions involving more than one enzymatic step. Metabolites written in brown were not analyzed in this study. Data are presented as the means ± standard deviation (n = 3). *ADP* adenosine diphosphate, *AMP* adenosine monophosphate, *ATP* adenosine triphosphate, *cAMP* cyclic adenosine monophosphate, *CDP* cytidine diphosphate, *CMP* cytidine monophosphate, *CTP* cytidine triphosphate, *GDP* guanosine diphosphate, *GMP* guanosine monophosphate, *GTP* guanosine triphosphate, *IMP* inosine monophosphate, *UDP* uridine diphosphate, *UMP* uridine monophosphate, *UTP* uridine triphosphate

With regards to purine metabolism, most metabolites except for two nucleobases (adenine and guanine) were found at their highest levels in the GlcNAc-grown cells (Fig. 5). However, for pyrimidine metabolism, the highest concentrations of UMP, UDP, and UTP were observed for the xylose-treated yeast, while CMP, CDP, CTP, cytidine, and cytosine levels were the highest in GlcNAc-grown cells. We also evaluated the levels of some deoxyribonucleotides (dATP, dCTP, and dTTP) in our CE-TOFMS analysis, but these metabolites were not detected in any of the conditions (Additional file 1: Table S1).

## Discussion

GlcNAc metabolism has been investigated in various microorganisms. In dimorphic yeasts, such as *C. albicans* and *Y. lipolytica*, GlcNAc is transported across the cell membrane and phosphorylated to GlcNAc6P by *N*-acetylglucosamine kinase (EC 2.7.1.59). Then, GlcNAc6P is deacetylated by *N*-acetylglucosamine-6-phosphate deacetylase (EC 3.5.1.25) to form GlcN-6-phosphate (GlcN6P), followed by deamination by glucosamine-6-phosphate deaminase (EC 3.5.99.6) to produce F6P [7, 11]. On the other hand, some bacteria, such as *Escherichia coli*, *Bacillus cadaveris*, *Streptococcus faecalis*, and *Vibrio cholerae,* have *N*-acetylglucosamine deacetylase (EC 3.5.1.33) activity and convert GlcNAc into GlcN [21, 22]. However, GlcNAc metabolism in *S. stipitis* has not been evaluated. In the present study, we investigated the metabolic response to GlcNAc in *S. stipitis* through a quantitative metabolomic analysis.

The levels of the GlcNAc derivatives (GlcN, GlcNAc6P, and GlcN6P) in GlcNAc-grown cells indicate that *S. stipitis* metabolizes GlcNAc into F6P through GlcNAc6P and GlcN6P similar to GlcNAc-assimilating dimorphic yeasts. Among the intermediates in the pathway from GlcNAc to ethanol, GlcNAc6P had the highest concentration (13.7 nmol/mg DCW), suggesting that deacetylation of GlcNAc6P to GlcN6P is a bottleneck step during anaerobic fermentation of GlcNAc. GlcNAc6P was detected not only in the GlcNAc-grown cells but also in glucose- and xylose-grown cells. Similarly, GlcN1P was also detected in all conditions. Chitin is an important component of the yeast cell wall and septum [23], and GlcNAc6P and GlcN1P are intermediates in the synthesis of chitin constituting the cell wall. In GlcNAc-containing medium, some of the assimilated GlcNAc might be directed to cell wall synthesis via GlcNAc6P and GlcN1P in *S. stipitis* cells. We also detected low concentration of GlcNAc (0.2 nmol/mg DCW) in the xylose-grown cells. Although it is unclear where this GlcNAc was brought from, it might be derived from chitin constituting the cell wall.

The data presented here also indicate a widespread effect of GlcNAc assimilation on metabolism in *S. stipitis*. In GlcNAc fermentation, *S. stipitis* produced acetate via the deacetylation of GlcNAc6P to GlcN6P. It has been reported that acetate indicates a prooxidant effect in yeast [24] and induces accumulation of reactive oxygen species (ROS) and programmed cell death of yeast cells [25]. In our CE-TOFMS analysis, GlcNAc-grown cells exhibited an approximately twofold higher GSSG/GSH ratio than glucose-grown cells. As the GSSG/GSH ratio is considered as an indicator of intracellular oxidative stress [26], these results indicate that GlcNAc-treated cells are exposed to higher levels of oxidative stress than glucose-grown cells. The elevated oxidative stress induced by acetate accumulation may also be a cause of the observed low cell growth in GlcNAc-containing medium. However, additional work is necessary to clarify this connection.

In our analysis, *S. stipitis* also generated ammonia during the deamination of GlcN6P to F6P in GlcNAc-containing medium. Ammonia is the most common nitrogen source for yeasts. In yeast cells, ammonia (NH_3_) is protonated, producing ammonium (NH_4_^+^) ion [27]. The ammonium ion is then assimilated by two primary reactions in the central nitrogen metabolism (CNM). The first reaction involves nicotinamide adenine dinucleotide phosphate-dependent glutamate dehydrogenase (NADPH-GDH), which uses α-ketoglutarate as the substrate and produces glutamate. The second reaction is catalyzed by glutamine synthase (GS), which uses glutamate as the substrate and produces glutamine [28]. Glutamate and glutamine generated via the CNM function as amino donors in most biosynthetic reactions in yeasts [29]. Further, ter Schure et al. [30] reported that the activity of NADPH-GDH and GS is regulated at the transcriptional and/or posttranscriptional levels in response to ammonia concentration in *S. cerevisiae*. The expression of the *GDH1* gene, which encodes NADPH-GDH, decreases with increasing ammonia concentration, and increased ammonia concentrations also decrease the relative activities of both NADPH-GDH and GS [30]. As a result of these regulations, the ammonia flux into the yeast biomass is maintained at a constant level regardless of the changes in ammonia concentration. In the present study, the concentrations of glutamine and glutamate in the GlcNAc-grown cells were comparable to those in glucose-grown cells despite the accumulation of ammonia in the former. Although transcriptomic and proteomic analyses are needed, our data indicate that *S. stipitis* may possess transcriptional and/or posttranscriptional regulatory mechanisms for ammonium assimilation in the CNM similar to those in *S. cerevisiae*, which function to prevent excessive assimilation of ammonia during GlcNAc fermentation.

**Fig. 6:**
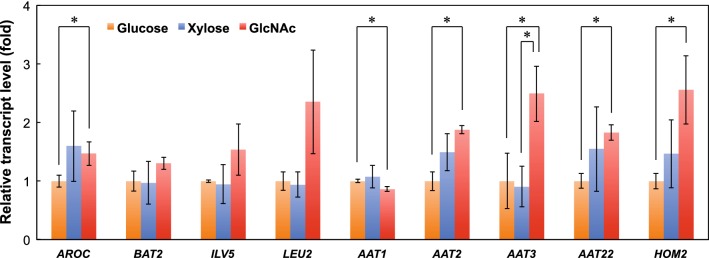
Comparison of transcript levels for genes involved in the amino acid biosynthesis in *S. stipitis* NBRC10063 after fermenting glucose (orange bars), xylose (blue bars), or GlcNAc (red bars) for 24 h. The relative transcript level of each gene is shown as a fold change in the mRNA level from the average of glucose. Data are presented as the means ± standard deviation (n = 3). Statistical significance was determined using Student’s *t* test (*p < 0.05)

Interestingly, unlike the similar levels of glutamine and glutamate, a wide range of other proteinogenic amino acids, including aromatic amino acids (phenylalanine, tyrosine, and tryptophan), branched-chain amino acids (leucine, isoleucine, and valine), and sulfur-containing amino acids (cysteine and methionine), were found at higher concentrations in the GlcNAc-grown cells. In a proteome analysis of *C. albicans* cells grown in GlcNAc or glucose, Kamthan et al. [12] reported that the expression of some enzymes involved in the biosynthesis of these amino acids, including chorismate synthase (EC 4.6.1.4), branched chain amino transferase, (EC 2.6.1.42), ketol acid reductoisomerase (EC 1.1.1.86), isopropyl malate dehydrogenase (EC 1.1.1.85), aspartate transaminase (EC 2.6.1.1), and aspartate semialdehyde dehydrogenase (EC 1.2.1.11), was significantly induced by GlcNAc. We performed the quantitative real-time PCR (qRT-PCR) analysis to observe the expression levels of nine genes encoding these enzymes in *S. stipitis* (Table 3). In the GlcNAc-grown cells, many of these genes showed enhanced expression and at least five genes (*AROC, AAT2, AAT3, AAT22,* and *HOM2*) demonstrated significantly higher transcript levels compared to that of the glucose-grown cells (*p* < 0.05) (Fig. 6). The qRT-PCR results were consistent with the proteome analysis of *C. albicans* described above. This result suggests that induction of expression of these genes is responsible for the increase in various amino acid concentrations in *S. stipitis* cells grown in GlcNAc.

**Table 3:** Selected genes for qRT-PCR in this study

Gene symbol	Locus tag	Gene description
*AROC*	PICST_90939	Chorismate synthase
*BAT2*	PICST_84005	Branched-chain amino acid transaminase
*ILV5*	PICST_78299	Mitochondrial ketol-acid reductoisomerase
*LEU2*	PICST_68561	3-Isopropyl malate dehydrogenase
*AAT1*	PICST_80440	Aspartate aminotransferase
*AAT2*	PICST_66059	Aspartate aminotransferase
*AAT3*	PICST_50446	Aspartate aminotransferase
*AAT22*	PICST_51039	Aspartate aminotransferase/Glutamic oxaloacetic transaminase
*HOM2*	PICST_89195	Aspartate-semialdehyde dehydrogenase

The effect of GlcNAc assimilation was particularly apparent on the levels of metabolites involved in purine and pyrimidine metabolism. Indeed, adenine, guanine, and cytosine nucleotide concentrations were the highest in GlcNAc-grown cells. Unlike the effects on amino acid biosynthesis, the induction of enzymes involved in purine and pyrimidine biosynthesis by GlcNAc or ammonia has not been reported previously. One possible cause for the increased levels of these nucleotides in GlcNAc-grown cells is the utilization of ammonia as the amino donor for glutamine-dependent amidotransferases, which play a role in the biosynthesis of a variety of purines and pyrimidine intermediates. For example, during the biosynthesis of inosine monophosphate (IMP) from R5P, two glutamine-dependent amidotransferases [amidophosphoribosyltransferase (EC 2.4.2.14) and phosphoribosylformylglycinamidine synthase (EC 6.3.5.3)] are involved. In addition, CTP synthetase (EC 6.3.4.2), which catalyzed the formation of CTP from UTP, is also a glutamine-dependent amidotransferase. It is also known that most glutamine-dependent amidotransferases utilize not only glutamine but also ammonia as the amino donor for their reactions [31]. Although further characterization of these amidotransferases in *S. stipitis* is required, it is likely that ammonia accumulated during GlcNAc fermentation is being utilized as an amino donor for these enzymes, facilitating the biosynthesis of adenine, guanine, and cytosine nucleotides in this yeast species.

GlcNAc is the monomeric constituent of chitin, which is the most abundant nitrogen-containing organic compound in nature [32]. Our data indicate that GlcNAc assimilation had a positive effect on the intracellular levels of a wide range of nitrogen-containing metabolites, including aromatic amino acids, branched-chain amino acids, and sulfur-containing amino acids, in *S. stipitis*. These amino acids are widely known to serve as precursors in the production of valuable nitrogen-containing compounds. For example, aromatic amino acids are the precursors of benzylisoquinoline alkaloids. Many of these nitrogen-containing compounds have highly desired pharmaceutical properties and, therefore, are associated with high market values [33]. Our results suggest that *S. stipitis* has the potential to be used as a host organism for the directed biosynthesis of these compounds from GlcNAc. However, this yeast consumed almost no acetate or ammonia derived from GlcNAc, leading to their accumulation in the medium. Therefore, in order to more efficiently utilize carbon and nitrogen derived from GlcNAc, genetic engineering to promote the utilization of acetate and ammonia will be necessary. Compared to *S. cerevisiae*, genetic engineering of *S. stipitis* is delayed due to its alternative codon system and frequent random (nonhomologous) integration [15]. Fortunately, genetic manipulation tools that can be applied to *S. stipitis* have greatly advanced in recent years [34]. Cao et al. [35], for example, recently developed a stable episomal plasmid and demonstrated high-efficiency gene knockout via the expression of CRISPR components on the plasmid. Thus, while additional work is necessary, the metabolomic data gathered in this study will be useful for designing effective genetic engineering strategies to develop novel *S. stipitis* strains for the production of valuable nitrogen-containing compounds from chitinous substrate-derived GlcNAc.

## Conclusions

In this study, we evaluated the metabolic responses to GlcNAc in *S. stipitis*. Anaerobic ethanol fermentation in media containing 50 g/L of GlcNAc and conventional carbon sources (glucose and xylose) was conducted and the intracellular metabolites were then extracted and quantified using CE-TOFMS to investigate the metabolic profile of the cells in each condition. Metabolomic analysis and subsequent PCA revealed increased accumulation of a wide range of nitrogen-containing metabolites, including amino acids, purines, and pyrimidines, during GlcNAc assimilation in *S. stipitis.* As many of these nitrogen-containing compounds are valuable in a variety of processes and products, *S. stipitis* could be utilized as a tool for their production using GlcNAc from chitin.

## Additional files

**Additional file 1: Table S1.** Concentration (nmol/mg DCW) of metabolites in *S. stipitis* NBRC10063 after 24 h fermentation on glucose, xylose, and GlcNAc and their factor loadings scores in principal component analysis.

**Additional file 2: Table S2.** Primers used in qRT-PCR.
